# Multi-Dimensional Quantum Capacitance of the Two-Site Hubbard Model: The Role of Tunable Interdot Tunneling

**DOI:** 10.3390/e25010082

**Published:** 2022-12-31

**Authors:** Andrea Secchi, Filippo Troiani

**Affiliations:** Centro S3, CNR-Istituto di Nanoscienze, I-41125 Modena, Italy

**Keywords:** quantum capacitance, quantum dots, quantum state discrimination

## Abstract

Few-electron states confined in quantum-dot arrays are key objects in quantum computing. The discrimination between these states is essential for the readout of a (multi-)qubit state, and can be achieved through a measurement of the quantum capacitance within the gate-reflectometry approach. For a system controlled by several gates, the dependence of the measured capacitance on the direction of the oscillations in the voltage space is captured by the quantum capacitance matrix. Herein, we apply this tool to study a double quantum dot coupled to three gates, which enable the tuning of both the bias and the tunneling between the two dots. Analytical solutions for the two-electron case are derived within a Hubbard model, showing the overall dependence of the quantum capacitance matrix on the applied gate voltages. In particular, we investigate the role of the tunneling gate and reveal the possibility of exploiting interdot coherences in addition to charge displacements between the dots. Our results can be directly applied to double-dot experimental setups, and pave the way for further applications to larger arrays of quantum dots.

## 1. Introduction

Semiconductor quantum dots (QDs) populated by charge carriers (electron or holes) are among the most studied systems for quantum computing [1,2,3]. A set of QDs, such as a QD array [4], is coupled to a classical circuit through a set of voltage gates [5], by means of which the experimenter can manipulate and measure the quantum state of the confined particles. In particular, the qubit readout can be efficiently performed within the gate reflectometry scheme, exploiting the capacitive coupling between the dots and the neighboring metal gates. The readout is performed by applying small voltage oscillations to one or more gates, and by estimating—from the reflected signal—the state-dependent change in the impedance of the resonant circuit [6,7,8,9,10]. When the frequency of the oscillation is slower than the charge carriers’ response to the varying potential (adiabatic regime), the impedance contributed by the QD(s) has a capacitive character [11]. In order to readout with high fidelity the state of particles in the dot, it is thus crucial to identify the regions in parameter space where such quantum capacitance (QC) presents large and state-dependent values. Typically, the largest values of the QC are achieved at the working points corresponding to transitions between different charge-stability regions, which correspond to voltage domains where the occupations of the QDs are relatively stable. At the boundaries between two such regions, the interplay between quantum tunneling on the one hand and QD potentials and Coulomb interactions on the other induces significant fluctuations in the QD occupations [12,13,14,15]. This can and has been exploited to implement the dispersive readout of spin qubits [16,17,18,19,20,21,22,23] and, more generally, for quantum-state discrimination [24].

In the adiabatic regime and in the presence of a single gate voltage *V*, the QC is related to the second derivative with respect to *V* of the relevant energy eigenvalue [25,26,27]. When M>1 gates are coupled to the system, the energy depends on *M* distinct voltage variables. This opens novel possibilities for the gate-reflectometry readout. The measured quantity, in fact, depends both on the working point in the *M*-dimensional voltage domain and on the coordinated oscillation applied to the gates, which identifies a probing direction in the same domain [5]. Specifying a probing direction amounts to fixing the relation between the oscillating voltages applied to the *M* gates during the measurement. Essentially, the QC peaks (for the given values of the applied voltages) represent suitable working points for detecting the quantum capacitance and, through its experimental estimate, inferring the quantum state. It is important, therefore, to have a theoretical knowledge of how the applicable gates determine the working points where the QC peaks are located, and how they can modulate the heights and widths of such peaks. In fact, as shown below, there are peaks in the QC that are not related to charge displacements between the dots, but rather to other, genuinely quantum features, such as the tunneling (interdot) coherences.

From a theoretical point of view, the multi-gate control of a nanostructure can be described in terms of a quantum capacitance matrix (QCM), corresponding to the Hessian matrix of an eigenenergy with respect to the gate voltages [28]. This formalism allows one to determine—for each given working point—the largest possible QC, defined by the largest-modulus eigenvalue. This is accessed by probing the QD array along a direction in the voltage space, defined by the corresponding eigenvector of the QCM. In Ref. [28], we developed a general approach to multidimensional quantum capacitance, and have applied our theory to a quantum-dot array, where the gates only control the minima of the QDs’ confining potentials. The purpose of the present work is to explore the scenario in which the gate that controls the tunneling barrier between the QDs can also be involved and contribute to the measured quantum capacitance. The physical system we refer to is represented by a double quantum dot (DQD), populated by two electrons and coupled to three gates: one for each electrostatically defined QD, and one modulating the interdot barrier. We thus focus on a two-site Hubbard model, which can be solved analytically, so as to yield the closed expressions of immediate application for the eigenenergies and for the eigenvalues and eigenvectors of the QCM.

In order to apply this theory to a real system, we relate the control parameters (gate voltages) to a Si/SiGe triple QD. The realistic ranges of parameters are obtained by referring to the values measured in that device. The analytical formulas that we provide, and which represent our main result, can, however, be applied to different devices as well. By examining the properties of several QCM-related quantities, we show that the great tunability of the tunneling allows one to shape the widths and heights of the QC peaks. The positions of the absolute QC peaks in voltage space lie close to those predicted by semiclassical considerations related to the charge movement between the QDs, in the regime where the tunneling is much smaller than the on-site repulsive interaction. By exploiting the exponential dependence of tunneling on the applied gate voltage, we push the parameters to and above the limits of such a regime, and we find new features (including relative peaks) in the QC profile that are related to tunneling coherences, rather than charge movement. Despite the fact that the model parameters yielding the largest values of coherence-related features lie in a domain where the Hubbard model loses its adherence to real systems, the general principle that the tunneling gate allows one to probe the tunneling coherence holds, and can be exploited for experimental measurements. Ultimately, the purpose of this work is two-fold: on the one hand, we intend to illustrate, within a specific and analytically solvable model, the potentialities of the multidimensional QC introduced in Ref. [28]; on the other hand, we point at the existence of a different physical regime, where the strength of interdot tunneling is comparable with other energy scales in the system and the QC has a different physical origin with respect to the known cases.

The remainder of this article is organized as follows. In Section 2, the concept of model quantum capacitance matrix (MQCM) is introduced; in Section 3.1, the effective model for the DQD is formulated and solved analytically; in Section 3.2, the analytical expressions for the MQCM are shown; in Section 3.3, the formulas for the total QC and its eigenvalues and eigenvectors are provided; in Section 4, the formulas derived in the previous Sections are numerically computed, and the general trends are discussed; in Section 5, we summarize our findings and mention possible future developments.

## 2. Model Quantum Capacitance Matrix

The QCM for a system in its *k*-th eigenstate is a matrix $C_{k;i,j}$, whose elements are defined by the second-order derivatives of the energy eigenvalue $E_{k}$ with respect to the gate voltages $V_{i}$ [28].

Any measurable QC is a linear combination of the elements of the QCM, and the coefficients of such a combination are determined by the direction (chosen by the experimenter) of the voltage oscillation in the *M*-dimensional voltage space. In other words, the measured QC can always be expressed as a weighted average of the eigenvalues of the QCM. At a given working point $V=(V_{1},\ldots ,V_{M})$, the QC eigenvalue with the largest modulus represents the maximum measurable value of the QC, and the direction of the voltage oscillation that should be applied in order to obtain such a value is given by the corresponding QC eigenvector [28].

From a theoretical point of view, the eigenenergies $E_{k}$ and the corresponding eigenstates are expressed in terms of the parameters entering the Hamiltonian, which defines the adopted model. This suggests the possibility of defining a model quantum capacitance matrix (MQCM), whose elements are given by the mixed second derivatives of the relevant energy eigenvalue with respect to the $M_{par}$ model parameters $p_{i}$:

(1) $$\begin{array}{c} C_{k;i,j}^{\mathrm{mod}}\equiv \frac{\partial^{2}E_{k}}{\partial p_{i}\partial p_{j}}. \end{array}$$

The model parameters corresponding to a given physical system are controlled by the applied gate voltages $V_{j}$. For a known functional dependence of the $p_{i}$ on the $V_{j}$, one can express the QCM as a function of the model quantum capacitance matrix through the equation

(2) $$\begin{array}{c} C_{k;i,j}=\sum_{\gamma ,\gamma^{\prime }=1}^{M_{\mathrm{par}}}\frac{\partial p_{\gamma }}{\partial V_{i}}\;C_{k;\gamma ,\gamma^{\prime }}^{\mathrm{mod}}\;\frac{\partial p_{\gamma^{\prime }}}{\partial V_{j}}\equiv \sum_{\gamma ,\gamma^{\prime }=1}^{M_{\mathrm{par}}}\alpha_{i,\gamma }\;C_{k;\gamma ,\gamma^{\prime }}^{\mathrm{mod}}\alpha_{j,\gamma^{\prime }}. \end{array}$$

The matrix α is, in general, rectangular if the number of tunable parameters $M_{\mathrm{par}}$ does not coincide with that of gate voltages *M*. The sector of α, which relates the plunger gates to the dots’ chemical potentials is the lever-arm matrix [29]. In the following, we consider a case where α is a square matrix, i.e., $M_{\mathrm{par}}=M$. Additionally, we assume for simplicity that the matrix is diagonal, i.e., that each model parameter depends on a different gate voltage. A similar situation is experimentally implemented by identifying the virtual gates: these are suitable combinations of the gate voltages that modify only one model parameter, while leaving the others unaffected [5,29]. In general, one can derive the α matrix for each device either experimentally or by carrying out a multiscale simulation of the device [30].

## 3. Double Quantum Dot Coupled to Three Voltage Gates: Analytical Expressions

### 3.1. Model Hamiltonian and Its Diagonalization

The considered system is represented by a DQD occupied by N=2 electrons and controlled by three gate voltages ($V_{1}$, $V_{2}$, and $V_{3}$), which control the shape of the confinement potential. In a simple picture, these three gates modulate the energy levels of the two QDs and the height of the barrier between them. More specifically, the two QDs are represented as the sites of a Hubbard model, with interdot tunneling amplitude *T* (assumed to be real for the sake of simplicity) and onsite energies $\epsilon_{1}$, $\epsilon_{2}$. The Hamiltonian is given by

(3) $$\begin{array}{c} \hat{H}=\sum_{i=1}^{2}\left(\epsilon_{i}{\hat{n}}_{i}+U{\hat{n}}_{i,↑}{\hat{n}}_{i,↓}\right)+T\sum_{\sigma }\left({\hat{c}}_{1,\sigma }^{†}{\hat{c}}_{2,\sigma }+{\hat{c}}_{2,\sigma }^{†}{\hat{c}}_{1,\sigma }\right)\;, \end{array}$$

where *U* is the onsite interaction energy, and ${\hat{n}}_{i,\sigma }={\hat{c}}_{i,\sigma }^{†}{\hat{c}}_{i,\sigma }$ is the number operator corresponding to the *i*-th dot and spin σ, while ${\hat{n}}_{i}=\sum_{\sigma }{\hat{n}}_{i,\sigma }$.

With reference to the terminology introduced in Section 2, here, the tunable model parameters are $\epsilon_{1}$, $\epsilon_{2}$, and *T*; equivalently, we will use $\epsilon \equiv \epsilon_{2}+\epsilon_{1}$ and $\delta \equiv \epsilon_{2}-\epsilon_{1}$ in place of the couple $\left(\epsilon_{1},\epsilon_{2}\right)$. These parameters are modulated by the physical gates $V_{1}$, $V_{2}$, and $V_{3}$ applied by the experimenter; instead, we assume that *U* is unaffected. Therefore, $M=M_{\mathrm{par}}=3$.

The Hamiltonian (3) can be analytically diagonalized. In the case of N=2, there are six eigenstates comprising three singlets and a triplet. It is a simple matter to show that the triplet energy is $E_{\mathrm{tr}}\left(\epsilon ,\delta ,T\right)=\epsilon$. The derivation of the three singlet eigenvalues is doable but more complicated, because it involves the diagonalization of a non-trivial 3×3 matrix. Using the resolving formula for a third-degree polynomial equation and some algebraic manipulations, we obtain

(4) $$\begin{array}{cc} \; & E_{-J}\left(\epsilon ,\delta ,T\right)=\epsilon +\frac{2}{3}\left[U-\sqrt{3\delta^{2}+12T^{2}+U^{2}}\cos\left(\theta \right)\right],\\ \; & E_{U}\left(\epsilon ,\delta ,T\right)=\epsilon +\frac{2}{3}\left[U-\sqrt{3\delta^{2}+12T^{2}+U^{2}}\sin\left(\theta -\frac{\pi }{6}\right)\right],\\ \; & E_{U+J}\left(\epsilon ,\delta ,T\right)=\epsilon +\frac{2}{3}\left[U+\sqrt{3\delta^{2}+12T^{2}+U^{2}}\cos\left(\theta -\frac{\pi }{3}\right)\right], \end{array}$$

where

(5) $$\begin{array}{c} \theta \left(\delta ,T\right)=\frac{1}{3}\arccos\left[\frac{U\left(-9\delta^{2}+18T^{2}+U^{2}\right)}{{\left(3\delta^{2}+12T^{2}+U^{2}\right)}^{3/2}}\right]. \end{array}$$

The subscripts in Equation (4) refer to the well-known values of the corresponding eigenvalues in the absence of on-site energies (ϵ=δ=0):

(6) $$\begin{array}{cc} \; & E_{-J}\left(0,0,T\right)=-J\equiv -\frac{\sqrt{U^{2}+16T^{2}}-U}{2},\;E_{U}\left(0,0,T\right)=U,\;E_{U+J}\left(0,0,T\right)=U+J, \end{array}$$

where *J* is the exchange energy. To our knowledge, the general solutions in the presence of arbitrary on-site energies (Equation (4)) and the related quantities that we will derive in the following are novel results that can be directly applied to any DQD which can be represented by a Hubbard model.

**Figure 1:**
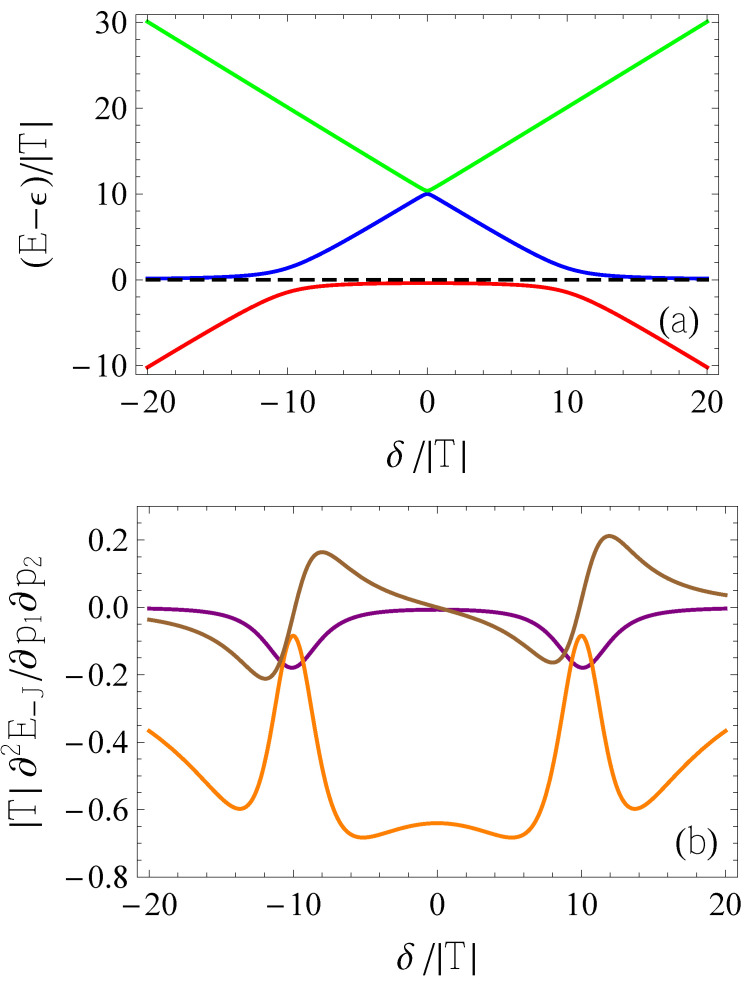
Relevant quantities related to the biased Hubbard model for the DQD occupied by two particles, as functions of δ/|T| and for U=10|T|. (**a**) Energy eigenvalues $E_{k}-\epsilon$ in units of |T| for k=−J (red), k=U (blue), k=U+J (green), and k=tr (black). (**b**) Nonzero elements of the MQCM for the ground-state energy, in dimensionless form given by $\left|T\right|\frac{\partial^{2}E_{-J}}{\partial p_{1}\partial p_{2}}$. The plotted curves correspond to different pairs $\left(p_{1},p_{2}\right)$: (δ,δ) (purple), (T,T) (orange), and (δ,T) (brown), under the assumption that T>0. If T<0, the brown curve should be multiplied by −1, while the other curves are unchanged.

At all values of the model parameters, the singlet with energy $E_{-J}$ is the ground state, while the first excited multiplet is the triplet. The eigenvalues (reckoned from ϵ, in units of |T|) are plotted in Figure 1a, as functions of δ/|T| and for U=10|T|. Relevant to the following discussion, the avoided crossings between the two lowest singlets (red and blue curves) at δ=±U correspond to the classical charge transitions (0,2)→(1,1) and (1,1)→(2,0), respectively, at δ=−U and δ=U. The particular U/|T| ratio used in Figure 1 was chosen for visualization purposes, but it can be significantly different, even by orders of magnitude, as discussed in the following sections. In particular, *T* strongly depends on the interdot distance and on the height of the tunneling barrier, which, in the present case, can be modulated by a gate voltage.

### 3.2. Model Quantum Capacitance Matrix

In terms of the model parameters (ϵ,δ,T), the MQCM for state *k* has three independent nonzero elements, namely

(7) $$\begin{array}{c} C_{k;2,2}^{\mathrm{mod}}=\frac{\partial^{2}E_{k}}{\partial \delta^{2}},\;C_{k;3,3}^{\mathrm{mod}}=\frac{\partial^{2}E_{k}}{\partial T^{2}},\;C_{k;2,3}^{\mathrm{mod}}=C_{k;3,2}^{\mathrm{mod}}=\frac{\partial^{2}E_{k}}{\partial \delta \partial T}\;. \end{array}$$

In fact, since the eigenenergies are linear in ϵ, all second derivatives involving ϵ vanish.

The analytical expressions for the quantities (7) related to the ground state (k=−J) are given by

(8) $$\begin{array}{c} \frac{\partial^{2}E_{-J}}{\partial p_{1}\partial p_{2}}\equiv \frac{8}{\sqrt{3\delta^{2}+12T^{2}+U^{2}}}\left[B_{p_{1},p_{2}}\frac{\cos(\theta )}{A}+C_{p_{1},p_{2}}\frac{U\sin(\theta )}{\sqrt{3}A^{3/2}}\right]\;\;\mathrm{with}\;\;p_{1},p_{2}\in \left\{\delta ,T\right\}, \end{array}$$

where

(9) $$\begin{array}{c} A={\left(\delta^{2}+4T^{2}\right)}^{3}-2\left(\delta^{4}-10\delta^{2}T^{2}-2T^{4}\right)U^{2}+\delta^{2}U^{4}, \end{array}$$

while $B_{p_{1},p_{2}}$ is a sixth-degree polynomial and $C_{p_{1},p_{2}}$ is an eighth-degree polynomial in the energies δ, *T* and *U*. Namely,

(10) $$\begin{array}{cc} \; & B_{\delta ,\delta }=-T^{2}\left[{\left(\delta^{2}+4T^{2}\right)}^{2}+\left(\delta^{2}+T^{2}\right)U^{2}\right],\\ \; & C_{\delta ,\delta }=T^{2}\left[T^{2}U^{4}-3\left(2\delta^{4}+\delta^{2}T^{2}-10T^{4}\right)U^{2}-2\left(5\delta^{2}-7T^{2}\right){\left(\delta^{2}+4T^{2}\right)}^{2}\right],\\ \; & B_{\delta ,T}=\delta T\left[{\left(\delta^{2}+4T^{2}\right)}^{2}-\left(\delta^{2}-2T^{2}\right)U^{2}\right],\\ \; & C_{\delta ,T}=\delta T[-\left(\delta^{2}+2T^{2}\right)U^{4}-6\left(\delta^{4}+2\delta^{2}T^{2}+10T^{4}\right)U^{2}\\ \; & \;\;\;+\left(7\delta^{2}-26T^{2}\right){\left(\delta^{2}+4T^{2}\right)}^{2}],\\ \; & B_{T,T}=-\delta^{6}-\delta^{4}\left(8T^{2}-2U^{2}\right)-\delta^{2}{\left(4T^{2}+U^{2}\right)}^{2}-4T^{4}U^{2},\\ \; & C_{T,T}=2[\delta^{2}\left(-2\delta^{2}+3T^{2}\right)U^{4}+2\left(2\delta^{6}-3\delta^{4}T^{2}+24\delta^{2}T^{4}+2T^{6}\right)U^{2}\\ \; & \;\;\;\;-\delta^{2}\left(2\delta^{2}-19T^{2}\right){\left(\delta^{2}+4T^{2}\right)}^{2}]. \end{array}$$

In Figure 1b, we plot the three inequivalent elements of the model quantum capacitance matrix (for U=10|T|). Interestingly, where the modulus of the diagonal element related to the bias (purple curve) presents the two maxima, the modulus of the element related to tunneling (orange curve) displays two pronounced minima. The different physical origins of the two terms, that correspond to the quantum capacitance measured by applying the oscillating voltage either to the bias or to the tunneling voltage, is thus reflected in the qualitatively different behaviors. The presence of a significant off-diagonal element (brown curve) further suggests that a maximal quantum capacitance might result from the simultaneous oscillations of the two voltages (see below).

### 3.3. Quantum Capacitance Matrix and Its Diagonalization

In order to compute the QCM, we now need to fix a relation between the control and model parameters. We assume that the physical gates were virtualized in such a way that each of them controls only one model parameter, according to

(11) $$\begin{array}{c} \epsilon_{1}=\alpha_{1}V_{1},\;\epsilon_{2}=\alpha_{2}V_{2},\;T=T_{0}e^{V_{3}/\beta }, \end{array}$$

where $\alpha_{1}$, $\alpha_{2}$, and β are positive. The exponential dependence of the tunneling on the corresponding gate voltage is justified by the fact that |T| is exponentially enhanced (suppressed) by lowering (increasing) the barrier height, which can be assumed to be linear in the applied voltage $V_{3}$. The accuracy of an exponential relation between $V_{3}$ and |T| has been experimentally demonstrated in Ref. [5]. With our convention, a large positive value of $V_{3}$ lowers the barrier and thus favors the interdot tunneling.

Under these assumptions, and for identical values of the two lever arms ($\alpha_{1}=\alpha_{2}\equiv \alpha$), one obtains the following QCM:

(12) $$\begin{array}{c} C_{k}\equiv \left(\begin{array}{ccc} X_{k} & -X_{k} & Y_{k}\\ -X_{k} & X_{k} & -Y_{k}\\ Y_{k} & -Y_{k} & Z_{k} \end{array}\right), \end{array}$$

where the matrix elements are given by

(13) $$\begin{array}{c} X_{k}\equiv \alpha^{2}\frac{\partial^{2}E_{k}}{\partial \delta^{2}},\;Y_{k}\equiv -\frac{\alpha T}{\beta }\frac{\partial^{2}E_{k}}{\partial \delta \partial T},\;Z_{k}\equiv \frac{T^{2}}{\beta^{2}}\frac{\partial^{2}E_{k}}{\partial T^{2}}. \end{array}$$

The matrix $C_{k}$ has a null eigenvalue, corresponding to the eigenvector $\left(1,1,0\right)/\sqrt{2}$ and related to the symmetric fluctuation of the on-site energies, assumed to linearly depend on the voltages $V_{1}$ and $V_{2}$. The remaining eigenvalues are:

(14) $$\begin{array}{c} C_{k;\pm }=X_{k}+\frac{Z_{k}}{2}\pm \sqrt{{\left(X_{k}-\frac{Z_{k}}{2}\right)}^{2}+2Y_{k}^{2}}. \end{array}$$

From the above expressions, it follows that $C_{k;-}\leq C_{k;+}$. Moreover, for the system ground state, $X_{-J}<0$ and $Z_{-J}<0$ (see Figure 1b). Therefore, one always has that $C_{-J;-}\leq C_{-J;+}\leq 0$ and that the QC eigenvalue with the largest modulus is $C_{-J;-}$. At the working points where the mixed partial derivative vanishes ($Y_{k}=0$), the QCM is block-diagonal, and the non-zero QC eigenvalues become

(15) $$\begin{array}{cc} \; & {\left.C_{k;-}\right|}_{Y_{k}=0}=\min\left(2X_{k},Z_{k}\right),\;{\left.C_{k;+}\right|}_{Y_{k}=0}=\max\left(2X_{k},Z_{k}\right). \end{array}$$

The normalized eigenvectors of $C_{k}$ corresponding to the non-zero eigenvalues can be written as

(16) $$\begin{array}{cc} \; & v_{k;-}=\left\{\begin{array}{c} \frac{\mathrm{sign}(Y_{k})}{\sqrt{{\left(C_{k;-}-Z_{k}\right)}^{2}+2Y_{k}^{2}}}\left(\frac{C_{k;-}-Z_{k}}{\sqrt{2}}\;,\;-\frac{C_{k;-}-Z_{k}}{\sqrt{2}}\;,\;\sqrt{2}Y_{k}\right),\;\mathrm{for}\;\;Y_{k}\neq 0\\ \frac{1}{\sqrt{2}}\left(1,-1,0\right),\;\mathrm{for}\;\;Y_{k}=0\;\mathrm{and}\;2X_{k}<Z_{k}\\ \left(0,0,1\right),\;\mathrm{for}\;\;Y_{k}=0\;\mathrm{and}\;Z_{k}<2X_{k} \end{array}\right.\;, \end{array}$$

and

(17) $$\begin{array}{cc} \; & v_{k;+}=\left\{\begin{array}{c} \frac{\mathrm{sign}(Y_{k})}{\sqrt{{\left(C_{k;+}-Z_{k}\right)}^{2}+2Y_{k}^{2}}}\left(\frac{C_{k;+}-Z_{k}}{\sqrt{2}}\;,\;-\frac{C_{k;+}-Z_{k}}{\sqrt{2}}\;,\;\sqrt{2}Y_{k}\right),\;\;\mathrm{for}\;Y_{k}\neq 0\\ \left(0,0,1\right),\;\mathrm{for}\;\;Y_{k}=0\;\mathrm{and}\;2X_{k}<Z_{k}\\ \frac{1}{\sqrt{2}}\left(1,-1,0\right),\;\mathrm{for}\;Y_{k}=0\;\mathrm{and}\;Z_{k}<2X_{k} \end{array}\right.\;. \end{array}$$

### 3.4. Singlet-Triplet Discrimination

The theoretical knowledge of the QC allows to discriminate between quantum states characterized by different QC matrices. A particular case of interest is the discrimination between ground states with different total spins. In the case at hand, as discussed above, the spin triplet has energy $E_{\mathrm{tr}}\left(\epsilon ,\delta ,T\right)=\epsilon$ and, therefore, its QCM is a null matrix. It follows that any QC measurement, at any working point, yields zero if the state of the system is a spin triplet. This is not altered by the inclusion of a magnetic field, which can only induce the energy splitting of triplet states, but no mixing between the triplet and the singlets.

Conversely, QC measurements on a singlet state reveal a very different scenario, as we will show in Section 4. The ground-state QCM is strongly dependent on both the working point and the direction of oscillation of the applied potential. The marked difference between the singlet and triplet QC matrices allows to experimentally discriminate between the two cases.

## 4. Numerical Results

We now derive from the above equations the main features of the three-gate QCM for the two-electron ground state in the DQD. In order to make our analysis as general as possible, it is convenient to refer to dimensionless quantities. In this system, the natural units of energy, voltages and QCs are $\left|T_{0}\right|$, $\left|T_{0}\right|/\alpha$, and $\alpha^{2}/\left|T_{0}\right|$, respectively. In the following, the dimensionless counterpart of a quantity *Q* is denoted as $\tilde{Q}$. Thus, for example,

(18) $$\begin{array}{cc} \; & \tilde{V_{i}}\equiv \frac{V_{i}}{\left|T_{0}\right|/\alpha },\;\tilde{\beta }\equiv \frac{\beta }{\left|T_{0}\right|/\alpha },\;\tilde{\delta }\equiv \frac{\left(V_{2}-V_{1}\right)}{\left|T_{0}\right|/\alpha }=\frac{\left(\epsilon_{2}-\epsilon_{1}\right)}{\left|T_{0}\right|},\\ \; & \tilde{\left|T\right|}\equiv e^{V_{3}/\beta }=e^{\tilde{V_{3}}/\tilde{\beta }},\;\tilde{U}\equiv \frac{U}{|T_{0}|},\;\tilde{C_{k}}\equiv \frac{\left|T_{0}\right|}{\alpha^{2}}C_{k}. \end{array}$$

### 4.1. Typical Ranges of Parameters

In Ref. [5], Mills and coworkers investigate Si/SiGe QDs separated by gated barriers; the tunneling amplitude between neighbor dots is demonstrated to be in an exponential relation with the barrier voltage. We refer to that work to deduce the appropriate orders of magnitude for the parameters of our model. Although the functional form that we adopt here for the dependence of *T* on $V_{3}$ is slightly different, it reproduces reasonably well the measured range of |T| in Ref. [5] with $|T_{0}|\approx 6\;\mu$eV, β≈46 mV, and $V_{3}$ ranging from 0 to several tens of mV, corresponding to $\tilde{\beta }\approx 10^{3}$ and ${\tilde{V}}_{3}$ up to some $10^{3}$.

The value of *U* cannot be estimated from Ref. [5], since that work considers transitions between single-particle states. Typical values, derived from previous calculations on Si and Ge DQDs [31], are of the order of some tens of meV. In particular, for an interdot distance of 16 nm, we find that U≈24 meV, corresponding to $\tilde{U}\approx 4000$. In the following, this is the value adopted for the onsite repulsion, while $\left(V_{2}-V_{1}\right)$ is varied in a range that allows to capture the *U*-dependent charge transitions at δ=±U.

### 4.2. Directly Measurable QCs: The Diagonal Terms of the QCM

As discussed in Ref. [28], all the elements of the QCM can be directly or indirectly experimentally estimated by suitable procedures. In particular, each diagonal element of the matrix, $C_{-J;i,i}$, is measured by applying an oscillation voltage only to gate *i*. Before discussing the QC eigenvalues, we show the dependence of such elements, and specifically $C_{-J;1,1}=C_{-J;2,2}$ and $C_{-J;3,3}$, on $\tilde{\delta }$ and ${\tilde{V}}_{3}$.

**Figure 2:**
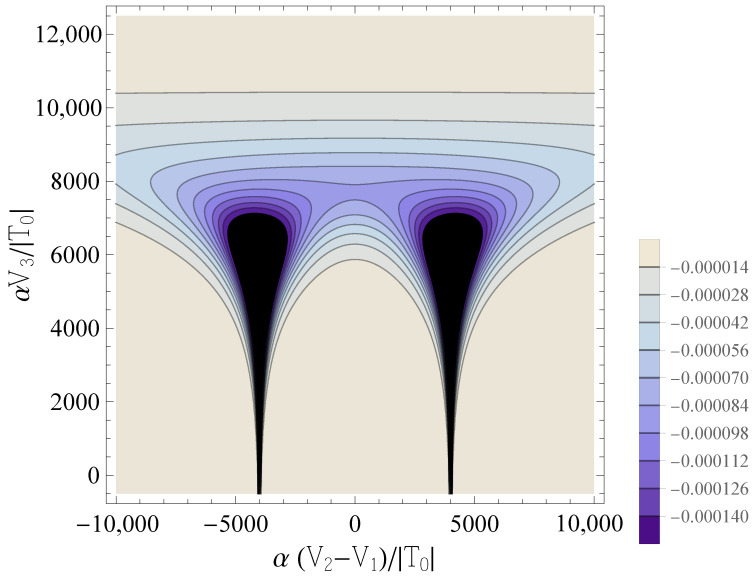
Contour plots of the diagonal element ${\tilde{C}}_{-J;1,1}$, as a function of $\tilde{\delta }$ and ${\tilde{V}}_{3}$, for $\tilde{\beta }=1000$ and $\tilde{U}=4000$. Darker colors correspond to more negative values of ${\tilde{C}}_{-J;1,1}$.

We start by investigating $C_{-J;1,1}$. We first observe that this quantity has a clear physical interpretation, which can be derived by the application of the Hellmann–Feynman theorem [28], namely,

(19) $$\begin{array}{c} C_{-J;1,1}=\alpha \frac{\partial {\langle {\hat{n}}_{1}\rangle }_{-J}}{\partial V_{1}}=\alpha \frac{\partial {\langle {\hat{n}}_{2}\rangle }_{-J}}{\partial V_{2}}=C_{-J;2,2}, \end{array}$$

i.e., it quantifies the variation of the occupation of dot *j* under small variations of the applied (virtual) gate potential $V_{j}$, which tunes the dot’s chemical potential. The behavior of $C_{-J;1,1}$ is reported in Figure 2. The contour lines corresponding to the highest absolute values of the QC are two drop-shaped domains which occur at $\tilde{\delta }\approx \pm \tilde{U}$ for a certain range of values of ${\tilde{V}}_{3}$—approximately ${\tilde{V}}_{3}<8000$ for the considered values of the parameters $\tilde{\beta }$ and $\tilde{U}$. When ${\tilde{V}}_{3}$ becomes large enough (${\tilde{V}}_{3}>8000$ in this case), the contour lines of the two drop-shaped domains merge into a single one. A further increase in ${\tilde{V}}_{3}$ results in a significant drop in the absolute value of the QC.

This is qualitatively explained in terms of the semiclassical picture developed in Ref. [28]: the QC peaks occur in regions of the parameters domain where the classical charge transitions [in this case, (0,2)→(1,1)→(2,0)] take place, and small variations in $V_{j}$ lead to large variations in the population of dot *j*. In these regions, the energies of the ground state and of the first excited singlet undergo an avoided crossing. The peaks’ height and width are related to the parameters describing such an avoided crossing, in particular to the ratio |T|/U. When ${\tilde{V}}_{3}$ is increased from the reference value 0 to higher values (i.e., the working point moves vertically from the bottom to the top of Figure 2), the amplitude of the avoided crossing gap, which is proportional to |T|, increases. Correspondingly, the height of the QC peak decreases, because the charge transition becomes smoother, i.e., the region in the parameter space where the occupation numbers of the two QDs are not well defined becomes larger. It should be noted that, for the parameters used in Figure 2, the value of ${\tilde{V}}_{3}\approx 8000$, where the drop-shaped contour lines merge, corresponds to $\left|\tilde{T}\right|\approx e^{8}$: the tunneling amplitude has thus been increased by a factor ≈3000 with respect to its value at ${\tilde{V}}_{3}=0$, and has reached a value that is comparable to the onsite repulsion $\tilde{U}$. Physically, this corresponds to a regime where the validity of the physical assumptions underlying the Hubbard model—the description of the double QDs in terms of weakly coupled site-localized states—becomes questionable.

**Figure 3:**
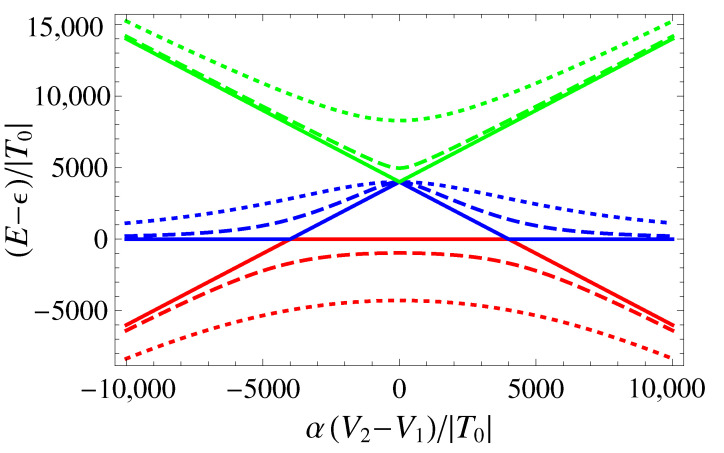
Singlet eigenenergies $E_{-J}$ (red), $E_{U}$ (blue), and $E_{U+J}$ (green), as functions of $\tilde{\delta }$, for $\tilde{\beta }=1000$, $\tilde{U}=4000$. The curves are computed for selected values of ${\tilde{V}}_{3}$, namely: ${\tilde{V}}_{3}=0$ (corresponding to $\left|\tilde{T}\right|=1$, solid curves), ${\tilde{V}}_{3}=7000$ ($\left|\tilde{T}\right|\approx 1097$, dashed), and ${\tilde{V}}_{3}=8000$ ($\left|\tilde{T}\right|\approx 2981$, dotted).

The effect of tuning the tunneling parameter manifests itself in the energy spectrum, as shown in Figure 3. The increase in $V_{3}$ leads to an exponential increase in |T|, which stretches the avoided-crossing gap between the ground state and the first-excited singlet from being negligible for $V_{3}=0$ (because of the large ratio U/|T|=4000) to being sizeable at ${\tilde{V}}_{3}=7000$ (corresponding to U/|T|≈3.65) and ${\tilde{V}}_{3}=8000$ (corresponding to U/|T|≈1.34). When $V_{3}$ is further increased, the three singlet energies tend to flatten, and the bias-induced charge transitions associated with the DQD structure are lost.

**Figure 4:**
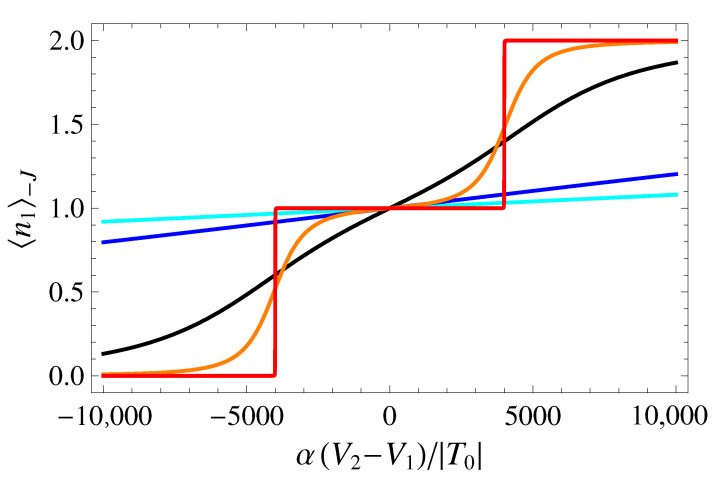
Average dot occupation ${\langle n_{1}\rangle }_{-J}$ as a function of $\tilde{\delta }$, for $\tilde{\beta }=1000$ and $\tilde{U}=4000$. Different colors correspond to the following values of ${\tilde{V}}_{3}$: 11,000 (cyan), 10,000 (blue), 7500 (black), 6000 (orange), 0 (red).

In order to visualize the charge configuration of the two dots, we compute the ground-state occupation numbers ${\langle n_{i}\rangle }_{-J}$ using the Hellmann–Feynman theorem, which yields [28]

(20) $$\begin{array}{c} {\langle n_{i}\rangle }_{k}=\frac{\partial E_{k}}{\partial \epsilon_{i}}=1+{\left(-1\right)}^{i}\frac{\partial E_{k}}{\partial \delta }, \end{array}$$

where the last equality is valid for the DQD. In Figure 4, we plot the occupation of dot 1 for the ground state, as a function of the bias $\tilde{\delta }$, for selected values of ${\tilde{V}}_{3}$. It can be seen that, for low values of ${\tilde{V}}_{3}$ (resulting in $T\approx T_{0}\ll U$), the occupation number changes abruptly with $\tilde{\delta }$, as expected within a semiclassical picture with U≫|T|. Instead, for values of ${\tilde{V}}_{3}$ yielding |T|≈U, the system undergoes a transition from a double QD to an effective single QD, as reflected in the plot of ${\langle n_{i}\rangle }_{-J}$ by the disappearance of the sharp charge transitions at $\tilde{\delta }=\pm \tilde{U}$, and by the flattening of the curve at the value 1, corresponding to the charge being evenly distributed between the two QDs.

**Figure 5:**
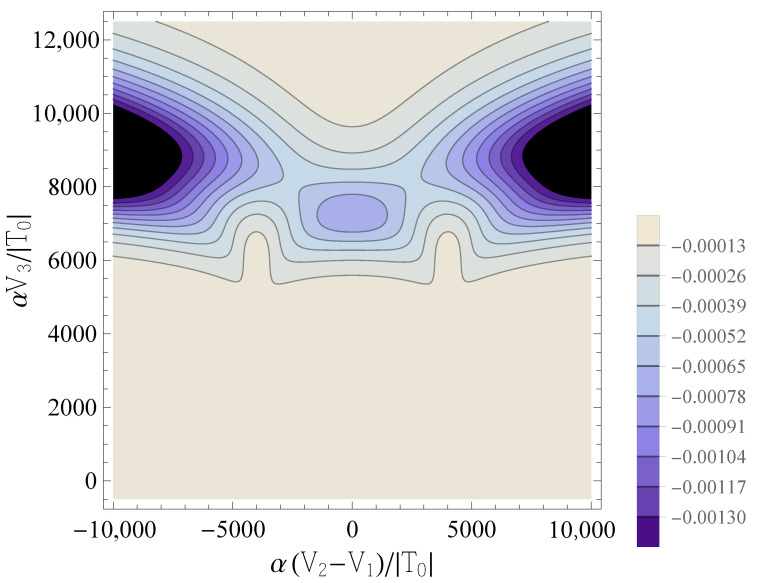
Contour plots of the diagonal element ${\tilde{C}}_{-J;3,3}$ as a function of $\tilde{\delta }$ and ${\tilde{V}}_{3}$, for $\tilde{\beta }=1000$ and $\tilde{U}=4000$. Darker colors correspond to more negative values of ${\tilde{C}}_{-J;3,3}$.

Figure 5 shows the contour plot of the other diagonal element of the QCM, namely $C_{-J;3,3}$, for the same set of parameters considered in Figure 2. We notice a strikingly different behavior compared to $C_{-J;1,1}$, due to the fact that $C_{-J;3,3}$ does not probe the charge transitions between the two QDs, as $C_{-J;1,1}$ does instead (see Equation (19)). In fact, from the Hellmann–Feynman theorem [28], it follows that

(21) $$\begin{array}{c} C_{k;3,3}=\frac{1}{\beta^{2}}T\left({\langle {\hat{h}}_{T}\rangle }_{k}+T\frac{\partial {\langle {\hat{h}}_{T}\rangle }_{k}}{\partial T}\right), \end{array}$$

where

(22) $$\begin{array}{c} {\hat{h}}_{T}=\sum_{\sigma }\left({\hat{c}}_{1,\sigma }^{†}{\hat{c}}_{2,\sigma }+{\hat{c}}_{2,\sigma }^{†}{\hat{c}}_{1,\sigma }\right) \end{array}$$

is the hopping operator in the Hubbard model. Therefore, just like the an oscillating bias voltage probes the dot occupations (Equation (19)), this QC measurement probes a combination of the tunneling coherence ${\langle {\hat{h}}_{T}\rangle }_{k}$ and of its derivative with respect to *T*. In the case considered herein, the highest peaks of $C_{k;3,3}$ occur at values of $V_{3}$ for which the description of the double dot in terms of the Hubbard model becomes questionable. Nevertheless, these features are interesting, since they indicate the possibility of obtaining peaks in the quantum capacitance that are qualitatively different from the ones related to the bias, and that have a physically different origin.

### 4.3. Directly Measurable QCs: The Largest Eigenvalue of the QCM

**Figure 6:**
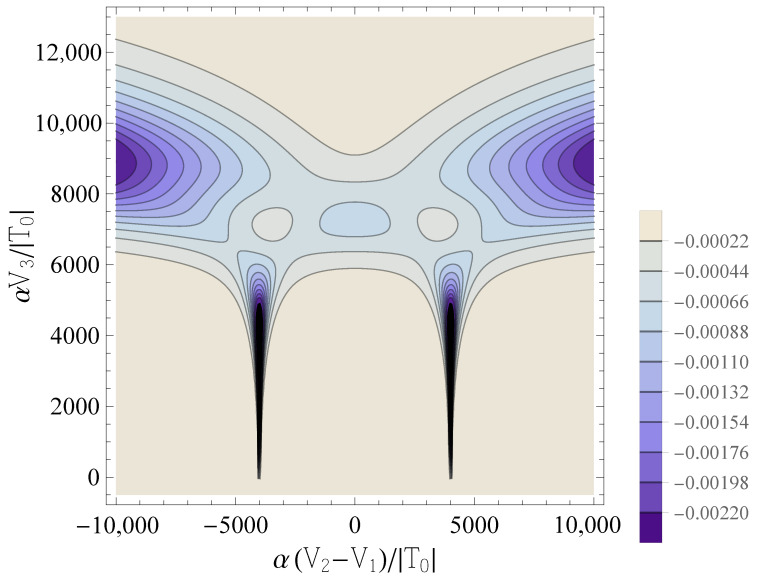
Contour plot of ${\tilde{C}}_{-J;-}$ as a function of $\tilde{\delta }$ and ${\tilde{V}}_{3}$ for $\tilde{\beta }=1000$ and $\tilde{U}=4000$. Darker colors correspond to more negative values of ${\tilde{C}}_{-J;-}$.

**Figure 7:**
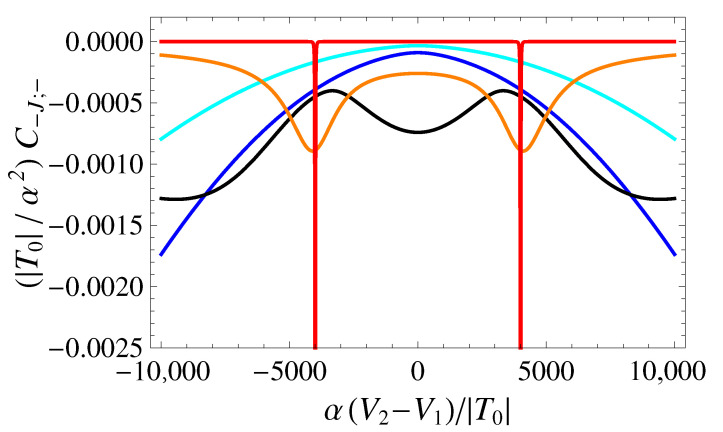
Eigenvalue $C_{-J;-}$ of the QCM as a function of $\tilde{\delta }$ for $\tilde{\beta }=1000$ and $\tilde{U}=4000$. Different colors correspond to different values of ${\tilde{V}}_{3}$, and specifically to: 11,000 (cyan), 10,000 (blue), 7500 (black), 6000 (orange), 0 (red).

If the oscillating voltage is applied along the direction in the voltage space defined by the eigenvectors of the QCM, then the measured QC is given by the corresponding eigenvalue. The most relevant outcome of the multidimensional quantum capacitance is represented by the eigenvalue with the largest modulus, corresponding to the largest measurable QC. This is obtained for the optimal direction in the voltage space, along which the signal should be applied. As above, we refer to the QC related to the Hamiltonian ground state, $C_{-J;-}$. In Figure 6, $C_{-J;-}$ is plotted as a function of $\tilde{\delta }$ and ${\tilde{V}}_{3}$, for $\tilde{\beta }=1000$ and $\tilde{U}=4000$ (the same values considered for the diagonal elements of the QCM). In Figure 7, we show the cross-sections of the contour plot of $C_{-J;-}$ at selected values of ${\tilde{V}}_{3}$.

From the comparison between the eigenvalue and the diagonal elements (Figure 2 and Figure 5), it is apparent that the peaks of $|C_{-J;-}|$ occur very closely to the peaks of $|C_{-J;1,1}|$ and $|C_{-J;3,3}|$. This suggests that, in the regions where the peaks of $|C_{-J,i,i}|$ are located, the *i*-th component of the QC eigenvector $v_{-J,-}$ dominates over the other two components. In other words, the main features that show up in the plot of the QC eigenvalue do not seem to result from significant mixing between the bias and tunneling voltages, but rather from the sum of the two diagonal contributions.

**Figure 8:**
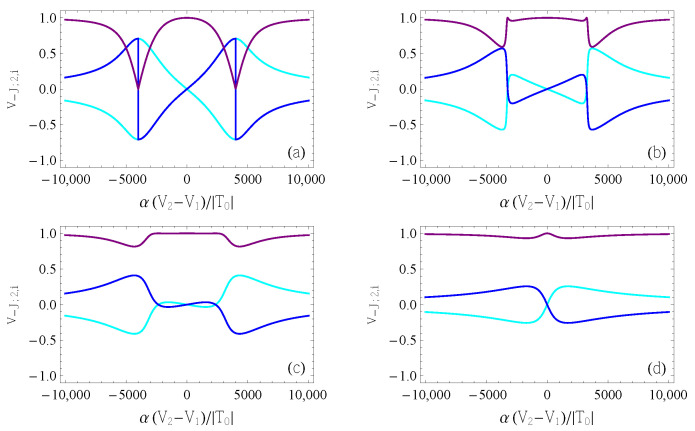
Components 1 (cyan), 2 (blue), and 3 (purple) of the eigenvector correspond to the lowest (most negative) eigenvalue of the QC matrix. Different colors distinguish the different components of the eigenvector. Different panels correspond to different values of ${\tilde{V}}_{3}$, as follows: (**a**) ${\tilde{V}}_{3}=0$; (**b**) ${\tilde{V}}_{3}=7200$; (**c**) ${\tilde{V}}_{3}=7500$; (**d**) ${\tilde{V}}_{3}=$ 10,000.

In order to quantitatively verify the above picture, we report the components of the QC eigenvector $v_{-J,-}$ as functions of $\tilde{\delta }$ and for selected values of ${\tilde{V}}_{3}$ (Figure 8). As already shown (Equation (17)), the components 1 and 2 are always opposites. Additionally, the peaks of their absolute values are located close to the charge transition points, $\tilde{\delta }=\pm \tilde{U}$. There, in the parameter range corresponding to weak interdot coupling, the dominant components in the eigenvector are those related to the bias (1 and 2). The eigenvector is instead dominated by the tunneling-related component (3) away from the charge transitions due to the exponential relation between the virtual and real tunneling gates. When the hopping is sufficiently large that the two QDs merge into a single one (e.g., Figure 8c,d), then component 3 dominates at all values of $\tilde{\delta }$, indicating that there are no charge transitions between the QDs. This is consistent with the scenario described by Figure 4.

## 5. Discussion and Conclusions

In conclusion, we applied the recently developed theory of multidimensional quantum capacitance to the case of a double QD, controlled by two bias gates and one tunneling gate. In particular, the use of the latter gate is shown to control the height and width of the peaks of the QC related to the change in charge configuration induced by the bias voltage. Besides, the tunneling voltage is responsible for the presence of additional peaks, with a qualitatively different physical origin. These peaks show up both in the relevant diagonal element of the QCM, and in its eigenvalue with largest modulus.

The analysis was performed within a biased Hubbard model, which allows the derivation of analytical solutions for all the relevant quantities, including the eigenvalues and eigenvectors of the QCM. On the other hand, the considered range of physical parameters partially exceeds the one where the Hubbard model represents an accurate description of the double-dot system. For these cases, more quantitative estimates will require the combination of the general approach for the derivation of the QCM with more accurate descriptions of the electron (or hole) eigenstates in the double quantum dot.

The procedure involves the determination of an effective Hamiltonian based on model parameters which enter the MQCM. The connection between the MQCM and the QCM also requires additional, device-specific information, determining the dependence of the model parameters on the real gates. This information might result from realistic multiscale device simulations, which quantitatively account for the coupling between the gate voltages on the one hand, and the tunneling and bias voltages on the other. In all cases, the possibility to quantitatively determine the optimal working points and directions of oscillation in the *M*-dimensional voltage space is expected to provide experimenters with more customizable tools for the manipulation and readout of quantum states.

## Data Availability

The data visualized and discussed in this work were obtained from the direct application of the analytical formulas derived and reported in Section 2 and Section 3.

## Abbreviations

The following abbreviations are used in this manuscript:

QC	Quantum Capacitance
QCM	Quantum Capacitance Matrix
MQCM	Model Quantum Capacitance Matrix
QD	Quantum Dot
DQD	Double Quantum Dot
